# Room-Temperature Self-Healing Conductive Elastomers for Modular Assembly as a Microfluidic Electrochemical Biosensing Platform for the Detection of Colorectal Cancer Exosomes

**DOI:** 10.3390/mi14030617

**Published:** 2023-03-07

**Authors:** Mei Wang, Zilin Zhang, Guangda Li, Aihua Jing

**Affiliations:** College of Medical Technology and Engineering, Henan University of Science and Technology, Luoyang 471003, China

**Keywords:** Ti_3_C_2_ MXene, room-temperature self-healing polyurethane, modular microfluidic, electrochemical biosensors, tetrahedral DNA nanostructure, colorectal cancer exosomes

## Abstract

Modular components for rapid assembly of microfluidics must put extra effort into solving leakage and alignment problems between individual modules. Here, we demonstrate a conductive elastomer with self-healing properties and propose a modular microfluidic component configuration system that utilizes self-healing without needing external interfaces as an alternative to the traditional chip form. Specifically, dual dynamic covalent bond crosslinks (imine and borate ester bonds) established between Polyurethane (PU) and 2-Formylbenzeneboronic acid (2-FPBA) are the key to a hard room-temperature self-healing elastomeric substrate PP (PU/2-FPBA). An MG (MXene/GO) conductive network with stable layer spacing (Al-O bonds) obtained from MXene and graphene oxide (GO) by in situ reduction of metals confers photothermal conductivity to PP. One-step liquid molding obtained a standardized modular component library of puzzle shapes from PP and MGPP (MG/PP). The exosomes were used to validate the performance of the constructed microfluidic electrochemical biosensing platform. The device has a wide detection range (50–10^5^ particles/μL) and a low limit of detection (LOD) (42 particles/μL) (S/N = 3), providing a disposable, reusable, cost-effective, and rapid analysis platform for quantitative detection of colorectal cancer exosomes. In addition, to our knowledge, this is the first exploration of self-healing conductive elastomers for a modular microfluidic electrochemical biosensing platform.

## 1. Introduction

Exosomes are essential in cancer’s early diagnosis and prognosis as potential biomarkers [1]. In contrast, efficient isolation methods and directly quantifiable ultra-sensitive exosome detection techniques remain challenging, especially in immediate diagnosis [2]. In recent years, microfluidic electrochemical biosensors have shown great promise in clinical and environmental analysis as a suitable assay for high-throughput screening, low limit of detection (LOD), real-time analyzing and small sample size [2,3,4,5]. The mainstream of current research is focused on paper-based microfluidic electrochemical sensors [3,4,5], but many shortcomings still need to be addressed. First, the inherent variability of the paper-based material reduces the accuracy of the hydrophobic barrier on the sensor and thus affects the measurement results. Second, the one-piece microfluidics make it very difficult to add new functions. Third, the sensor is easily damaged by bending and can only be used as a disposable product. These difficulties may be overcome using injection-moldable self-healing conductive elastomers for modular microfluidics.

The main challenges for existing modular microfluidic applications are accurate alignment and tight sealing to ensure leak-free and fluid interoperability between assembled modules [6]. Standardized, modular microfluidic components can be manufactured on-demand, relying on three-dimensional printing technology [7,8]. However, it relies heavily on interface technologies such as magnetic interfaces and O-rings [9,10]. Until now, self-healing elastomers have yet to be used on a large scale for modular microfluidics. Their excellent mechanical strength and room temperature self-healing capability are challenging to balance [11]. Moreover, the inability to withstand pressure can lead to the collapse and blockage of fluid channels [12]. Polyurethane (PU) elastomers are among the most versatile industrial polymers, and the introduction of multiple dynamic bonds in monomers has been proposed to create high-performance self-healing materials [11]. However, polymers with complex functions and structures are often not produced on a large scale, thus limiting practical applications [13]. Therefore, developing a low-cost, easy-to-synthesize self-healing elastomer is imperative.

Introducing conductive fillers into polymer networks is a common way to achieve conductive elastomers. MXene is a novel nanomaterial with an intact layer of metal atoms and large surface functional groups on its surface [14,15]. Like other two-dimensional materials, the strong van der Waals forces present between the lamellae cause irreversible agglomeration and overlap [15]. In recent years, many scholars believe that the three-dimensional porous structure formed by the rigid MXene and the flexible graphene oxide (GO) can improve the electrochemical properties. Chemical methods such as hydrothermal methods, heat treatment, and reagent reduction must usually be performed at low temperatures or under protective gas [16,17,18]. Otherwise, MXene oxidation and degradation will occur. Although the physical method avoids the above-mentioned complicated steps, the mechanical mixing and the self-assembly are not considered for the poor stability of MXene [19,20,21]. Therefore, MG’s (MXene/GO) rapid and straightforward synthesis at room temperature is still a significant challenge.

**Scheme 1:**
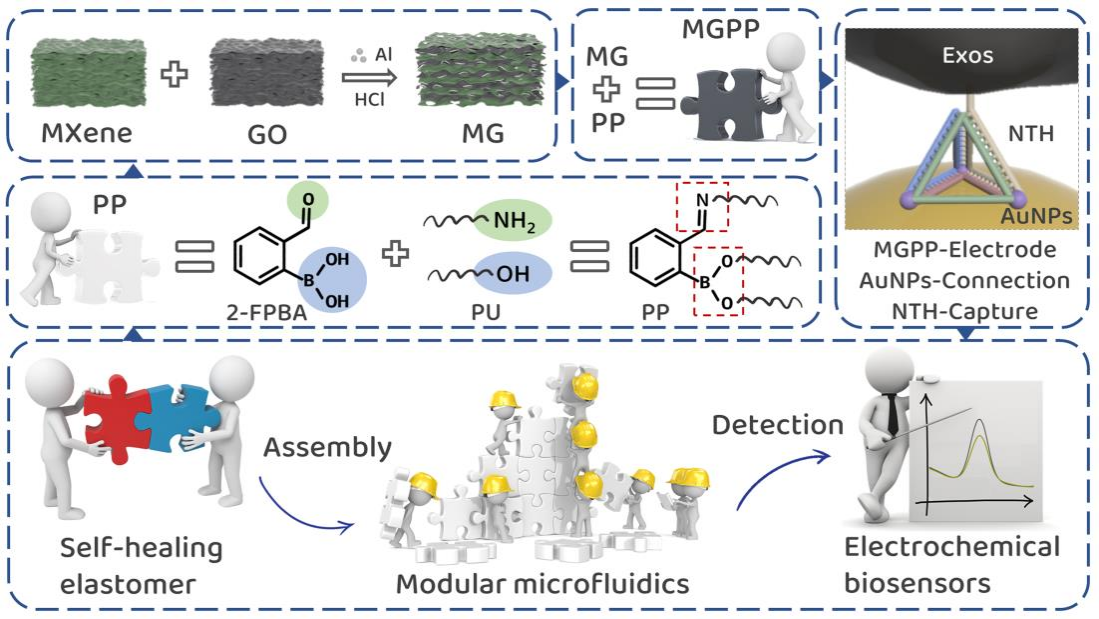
Experimental idea diagram of self-healing conductive elastomer for modular microfluidic electrochemical biosensing platform to detect colorectal cancer exosomes.

Herein, we demonstrate a conductive elastomer with self-healing properties and propose a new strategy for combining modular microfluidic components to achieve a microfluidic platform without complex interface technologies. Specifically (Scheme 1), PP (PU/2-FPBA) is covalently cross-linked by primary amine and hydroxyl groups in PU and aldehyde and boronic acid bonds in 2-Formylbenzeneboronic acid (2-FPBA), respectively, and the highly dynamic and reversible imine and boronic ester bonds make PP have excellent self-healing ability. MG obtained by spontaneous adsorption and layer-by-layer assembly of MXene and GO in the presence of Al powder has larger and more stable layer spacing, which results in better electrochemical properties of MG. A microfluidic electrochemical biosensing platform was constructed by selecting several base and functional modules obtained by injection molding with the help of precisely positionable puzzle shapes and excellent self-healing ability. MG was added to PP as a photothermally conductive filler to obtain MGPP (MG/PP). MGPP can be used as a self-supporting electrode for highly sensitive detection of colorectal cancer exosomes with the help of tetrahedral DNA nanostructure (TDN), which acts as a recognition molecule. In addition, to our knowledge, this is the first exploration of self-healing conductive elastomers as a modular microfluidic electrochemical biosensing platform through modular assembly.

## 2. Materials and Methods

### 2.1. Materials and Chemicals

The water used was triple distilled water. Unless otherwise stated, all chemicals were used without further purification. Titanium aluminum carbide powder (Ti_3_AlC_2_) and lithium fluoride (LiF) was produced by Xiyan New Material Technology Co., Ltd. (Shandong, China). Al power was produced by Pantian Powder Material Co., Ltd. (Shanghai, China). PU, 2-FPBA were purchased from Macklin Biotech Co., Ltd. (Shanghai, China). DNA sequences were purchased from Sangon Biotech Co., Ltd. (Shanghai, China).

### 2.2. Preparation of MXene

LiF (1.6 g) was added to HCl solution (20 mL, 12 M) and stirred for 10 min. Ti_3_AlC_2_ (1 g) was gradually added at 35 °C, and the solution was stirred for 24 h. The solution was then washed by centrifugation several times at 3500 rpm. Among them, the upper layer of liquid gradually became turbid, which indicated that MXene began to delaminate. After centrifugation to a pH of about 6, the solution was sonicated in an ice-water bath for 1 h. Subsequently, the supernatant was collected by centrifugation at 3500 rpm for 30 min and freeze-dried, and the resulting monolayer MXene was obtained.

### 2.3. Preparation of GO

Graphite powder (2.5 g) and NaNO_3_ (1.25 g) mixture were poured into 57.5 mL of H_2_SO_4_ in an ice bath and stirred continuously for 30 min. After that, KMnO_4_ (7.5 g) was added slowly in batches and stirred continuously for 30 min. After warming up to 35 °C and stirring for 2 h, water (120 mL) was added slowly in batches and then warmed up to 70 °C and stirred for 1 h. Water (250 mL) and 30% H_2_O_2_ (5 mL) were, in turn, added and left for 6~8 h. This was washed several times until the supernatant was tested with BaCl_2_ without white precipitate, and GO could be obtained.

### 2.4. Preparation of MG

The MG was obtained by adding Al powder (0.3 g) to a colloidal suspension of GO (1.5 mg) and Ti_3_C_2_ (28.5 mg) by shaking vigorously, washing off the excess Al powder with HCl, and freeze-drying.

### 2.5. PP and MGPP Preparation

The self-healing elastomer PP was prepared by mixing conventional PU and 2-FPBA in a 10:1.5 mass ratio in equal volumes. In contrast, the self-healing conductive elastomer MGPP was formed by mixing MG, carbon black, and PP in an 8:1:1 weight ratio. The self-healing effect was clearly shown by adding different colors of solid color essence and recorded using a digital camera. After a piece of PP of 1 cm × 1 cm size was wholly cut off, the healing process of PP was observed through the microscope, and the time was recorded. Two pieces of MGPP of 1 cm × 1 cm size were aligned, and their photothermal conversion ability was observed using a NIR laser, and a thermal imaging camera recorded the temperature.

### 2.6. Preparation of Modular Components

The module of the puzzle shape is 1.3 cm long and 1 cm wide, and the diameter of the circular piercing point is 0.25 cm. The entire mold was covered with PP and MGPP just above the mold surface height. The covered modular mold was then placed under a vacuum until the surface was stationary and free of air bubbles. After drying at room temperature, the modular components were slowly extracted from the mold surface by applying moderate pressure. The components could be quickly extracted without damaging the mold or the components. Ink was injected to check for leaks.

### 2.7. Preparation of TDN

The ssDNA was dissolved in TM buffer to a final concentration of 100 μM. The monomers that formed the tetrahedra were first treated with 10 mM tris (2-carboxyethyl) phosphine (TCEP) for 2 h at room temperature. Then, the single chains used to synthesize the DNA tetrahedra were mixed in equal proportions in TM Buffer. The tetrahedral nanostructures were formulated to a final concentration of 1 μM. The formulated samples were self-assembled to form TDN by continuing in a PCR instrument at 95 °C for 10 min, then rapidly cooled down to 4 °C and continued at 4 °C for more than 10 min. The DNA sequences were: A: ACATTCCTAAGTCTGAAACATTACAGCTTGCTACACGAGAAGAGCCGCCATAGTACTGACCACGAGCTCC; B: SH-TATCACCAGGCAGTTGACAGTGTAGCAAGCTGTAATAGATGCGAGGGTCCAATAC; C: SH-TCAACTGCCTGGTG-ATAAAACGACACTACGTGGGAATCTACTATGGCGGCTCTTC; D: SH-TTCAGACTT-AGGAATGTGCTTCCCACGTAGTGTCGTTTGTATTGGACCCTCGCAT; Epcam aptamar: CACTACAGAGGTTGCGTCTGTCCCACGTTGTCATGGGGGGTTGGCCTGCT-AATGGAGCTCGTGGTCAG.

### 2.8. Preparation of AuNPs

A volume of 10 mL of 38.8 mM trisodium citrate was added to a boiling 100 mL solution of 1 mM HAuCl_4_. The boiling state was maintained and stirring was continued for 15 min. The solution was cooled naturally to room temperature to obtain a burgundy gold sol.

### 2.9. Preparation of Electrochemical Biosensors

The electrochemical biosensing chip comprises three inlet modules, an electrochemical detection unit and an outlet module. Among them, a single inlet and two Y-shaped double inlets correspond to 1 mg/mL AuNPs, 1 μM TDN, 1 μM Epcam aptamer, different concentrations of exosome solutions, and phosphate buffered saline (PBS) solution infusion, respectively. The solutions were introduced one by one into the chambers within the electrochemical module through different inlet channels and incubated separately for 1 h at room temperature. The obtained assembled electrochemical biosensor is ready for the quantitative detection of exosomes.

### 2.10. Material Characterization

XRD (Panalytica, Hol-land) was used to determine the crystal structure of the MG. Raman spectra were obtained on a Renishaw inVia confocal spectrometer by using 633 nm line excitation. The morphology of MG was studied by TEM (FEI, Tecnai G2 F30). An X-ray photoelectron spectrometer Thermo escalab 250Xi tested XPS. FT-IR was performed on a Nicolet IS10 using the KBr press method for MG and attenuated total refraction (ATR) mode for PP. The photothermal conversion capability of MGPP was monitored using a Laserwave LWIRL NIR laser, and the temperature was recorded using a thermal imaging camera (Testo860).

### 2.11. DFT Calculations

All calculations were performed by using the Cambridge sequential total energy (CASTEP) package of programs, using the on-the-fly generated (OFTG) ultra-soft pseudopotential and the Perdew-Burke- Ernzerhof (PBE) exchange-correlation generalization under the generalized gradient approximation (GGA). The energy cutoff was set to 750 eV. Convergence tolerance were 0.01 eV/Å (force), 5.0 × 10^−7^ eV/atom (energy) and 5.0 × 10^−4^ Å (displacement). The vacuum space of 15 Å was sufficient to avoid errors due to periodic boundaries. The adsorption energy ΔE is defined by the equation ΔE = E_M,Al_ − (E_M_ + E_Al_), where E_Al_ represents the energy of Al, E_M_ represents the energy of MXene or GO containing surface groups, and E_M,Al_ represents the total energy of M after adsorption with Al. Differential charge density reveals the electron accumulation and depletion.

### 2.12. Electrochemical Measurements

All electrochemical measurements were performed on an electrochemical workstation (CHI 660e). Electrochemical properties were tested in a 0.5 mM [Fe(CN)_6_]^3−/4−^ solution containing 0.1 M KCl under a three-electrode system. Ag/AgCl and platinum wire electrodes were used as reference and counter electrodes. Cyclic voltammetric (CV) and electrochemical impedance spectroscopy (EIS) measurements during the stepwise modification of the biosensor were recorded at a sweep speed of 10 mV/s and in the frequency range of 0.1–100 kHz, respectively. According to the equation log(i) = blog(v) + loga, calculating the slope can distinguish the electrochemical behavior. Moreover, the contribution of each component to the current can be further quantified by i(v) = k_1_v + k_2_v^1/2^. Differential pulse voltammetry (DPV) measurements were performed for exosomes at concentrations of 0, 50, 10^2^, 5 × 10^2^, 10^3^, 5 × 10^3^, 10^4^, 5 × 10^4^, and 10^5^ particles/μL in the potential range of −0.1 V to 0.6 V, respectively. The sensing platform’s sensitivity, smoothness, and reproducibility were tested using exosome concentrations of 10^3^ particles/μL.

## 3. Results and Discussion

**Figure 1:**
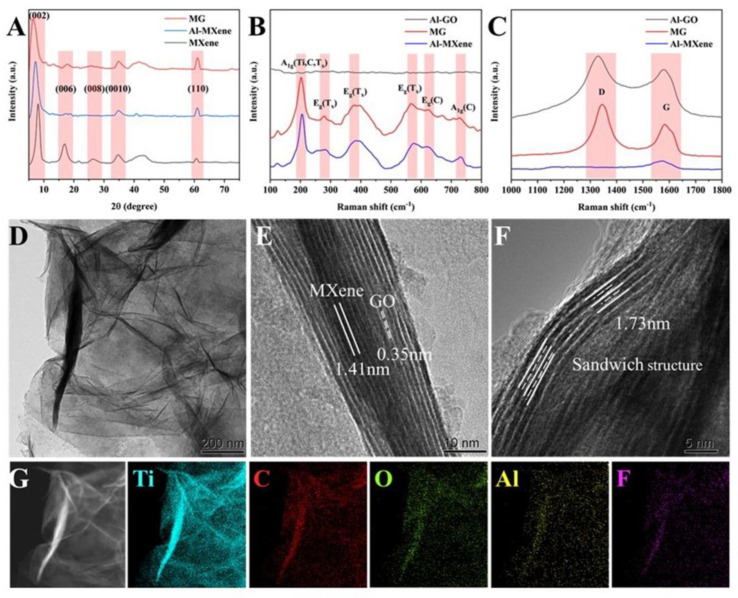
Successful synthesis of MG. (**A**) XRD, (**B**,**C**) Raman, (**D**) TEM, (**E**,**F**) HR−TEM, and (**G**) EDS.

MXene was formed by etching of the Al atomic layer of the Ti_3_AlC_2_ MAX phase [14]. The disappearance of the peak at 39.1° in the X-ray diffraction (XRD) diagram (Figure 1A) indicates the successful synthesis of Mxene. Among them, 8.2°, 16.8°, 26.3°, 34.7°, and 60.7° indicate the (002), (006), (008), (0010), and (110) crystal planes of MXene, respectively. Meanwhile, Al-MXene and MG exhibit similar shapes to MXene in XRD, and no new material is generated [22]. In addition, it can be observed in Figure 1A that the (002) diffraction peak has been shifted to the left, decreasing from 8.2° for pure MXene to 7.2° for Al-MXene and 6.1° for MG, indicating an increase in the layer spacing from 1.07 nm to 1.45 nm. The larger layer spacing of MG is expected to provide more electroactive sites for electrolyte ions. In comparison, the (002) peak of MG is wider than that of MXene, demonstrating that the restacking of MXene is prevented [15]. Figure 1B,C show the Raman spectra diagram of Al-Mxene, MG, and Al-GO, respectively. For Al-Mxene (Figure 1B), 206 cm^−1^ corresponds to the symmetric out-of-plane vibration (A_1g_) of Ti, C, and the surface group (T_x_); 282 cm^−1^, 384 cm^−1^, and 576 cm^−1^ can be attributed to the in-plane vibration (E_g_) of T_x_; while 618 cm^−1^ and 731 cm^−1^ indicate E_g_ and A_1g_ of C, respectively. The MG in the Raman diagram exhibits a Raman pattern similar to Al-Mxene, and the larger layer spacing shifts the A_1g_ (C) peak toward the large offset [22,23]. The presence of rGO causes the D (1345 cm^−1^) and G (1582 cm^−1^) bands of the MG to exhibit similar growth to Al-GO [21] (Figure 1C), verifying the complexation in MG.

In the transmission electron microscopy (TEM) image (Figure 1D), many irregular folds can be observed on the surface of the MG, which is quite different from the rigid MXene. High resolution transmission electron microscopy (HR-TEM) (Figure 1E) can observe lattice spacing of 1.41 nm and 0.35 nm, corresponding to the (002) crystal plane of MXene and rGO, respectively [24,25]. A sandwich structure (1.73 nm) shown in Figure 1F may be attributed to the embedding of GO between MXene layers, demonstrating the heterogeneous assembly of MXene. This structure could inhibit further stacking of MXene. Energy dispersive spectroscopy (EDS) plots (Figure 1G) show a uniform distribution of Ti, C, O, Al, and F throughout the field of view, indicating the coexistence of MXene and rGO and the cross-linking effect of Al^3+^ between MXene and rGO nanosheets.

**Figure 2:**
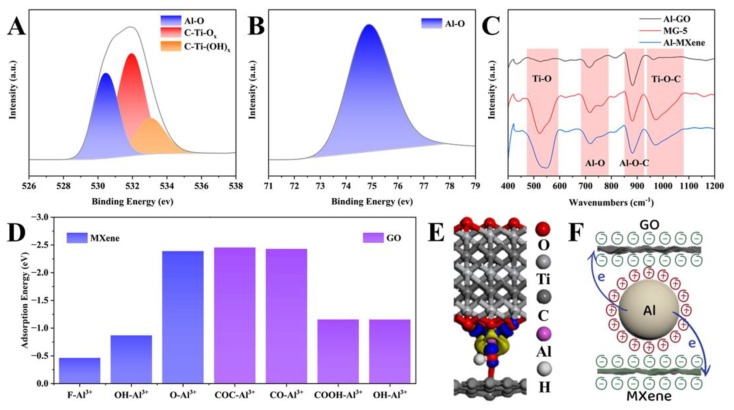
Cross-linking mechanism of MG. XPS of O 1s (**A**), Al 2p (**B**), and (**C**) FT−IR. (**D**) Adsorption energy. (**E**) Differential charge density maps. The blue and yellow areas represent the electron accumulation and depletion, respectively. (**F**) The formation process of MG.

We believe there is a strong interaction between Al^3+^ and MXene and GO nanosheets to fix the adjacent nanosheets and produce a stable intercalation distance. We first tested X-ray photoelectron spectroscopy (XPS) to verify this conjecture. In O 1s (Figure 2A), the peaks located at 533.1 eV, 531.9 eV, and 530.4 eV correspond to C-Ti-(OH)_x_, C-Ti-O_x_, and Al-O bonds [26], respectively. In Al 2p (Figure 2B), the peak at 74.85 eV also corresponds to Al-O bonds [27], indicating that Al^3+^ tends to bind to oxygen-containing functional groups on the surfaces of MXene and GO nanosheets. Similar Fourier transform infrared spectroscopy (FT-IR) of Al-Mxene, MG, and Al-GO are shown in Figure 2C. Among them, the peaks located at 522 cm^−1^, 972 cm^−1^, and 882 cm^−1^ correspond to the formation of the Ti-O bond, Ti-O-C bond, and Al-O-C bond, respectively, while the peak at 713 cm^−1^ further demonstrates the formation of Al-O bond [28]. To gain insight into the interaction between Al^3+^ and the two nanosheets, the adsorption of Al^3+^ on the nanosheet surface was investigated by density flooding theory (DFT) calculations. The DFT results (Figure 2D) revealed that the binding energies of Al^3+^ on the -F, -OH, and -O capped MXene nanosheets were −0.46 eV, −0.87 eV, and −2.38 eV, respectively. At the same time, the order of the stability of Al^3+^ adsorption on GO nanosheets containing multiple groups is -COC (−2.45 eV) > -CO (−2.43 eV) > -COOH (−1.15 eV) ≈ -OH (−1.15 eV). These two results indicate that the oxygen group located on the surface of MXene nanosheets [27] and the epoxy group located on the surface of GO nanosheets [29] are the most stable adsorption sites for Al^3+^. The differential charge density map (Figure 2E) reveals electrons’ depletion from Al and electrons’ accumulation from the oxygen-containing group during the interaction between Al and the two nanosheets. The charge transfer from Al to the oxygen-containing group is critical evidence for forming the Al-O bond, stabilizing the d-spacing.

Thus, MG’s formation process and mechanism are presumed as follows (Figure 2F). The continuous ionization of Al powder releases many electrons and Al^3+^ [30]. Among them, the electrons participate in the reduction reaction of MXene and GO (MXene + GO + ne^−^ + nH^+^ →rMXene + rGO + nH_2_O) [31]. At the same time, Al^3+^ will cause spontaneous adsorption and layer-by-layer assembly of MXene and GO nanosheets through electrostatic attraction [32]. Notably, in contrast to random physical mixing, the strong interactions inhibit the self-accumulation of rGO or MXene nanosheets.

**Figure 3:**
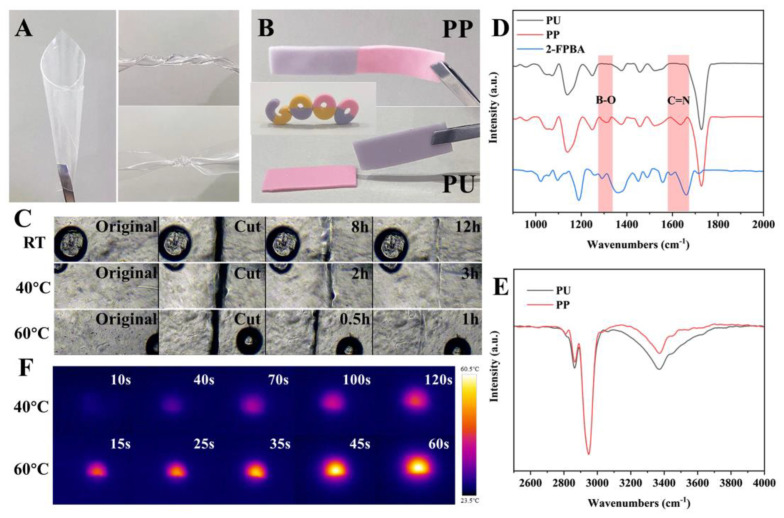
Self-healing properties of PP. (**A**) PP can be bent, twisted and knotted. (**B**) PP can heal itself at room temperature, while PU cannot. (**C**) Optical microscope observation of the self-healing ability of PP at different temperatures. (**D**,**E**) FT−IR. (**F**) Infrared thermal images of MGPP during warming up.

PP from 2-FPBA and PU can be made into any shape by changing the mold. Benefiting from the excellent properties of PU, PP also exhibits good elasticity, as shown by the fact that PP can be bent, twisted, and knotted (Figure 3A). To evaluate the self-healing properties, PU and PP were stained to facilitate the differentiation of the interfaces. While PU cannot self-heal at room temperature, PP has no visual interface at the fracture and remains intact even under stretching [33] (Figure 3B). The self-healing process of transparent PP was visualized by optical microscopy [34] (Figure 3C). The boundary between PP became blurred, and the interconnected area became continuous [33]. However, healing traces are challenging to remove at room temperature, as the limited mobility of polymer chain segments can limit the interpenetration of interfacial macromolecules [35]. In contrast, high temperature causes the polymer molecular chains to exhibit enhanced mobility, which promotes crack healing. Therefore, healing time is significantly reduced from 12 h at room temperature to 1 h at 60 °C [34].

The critical pathway for self-healing is the dual dynamic covalent cross-linking established between 2-FPBA and PU [36,37]. FT-IR verified the generation of imine and borate ester bonds. The significant decay [38] of PP at 3250 cm^−1^ (Figure 3E) was attributed to (1) the Schiff base reaction between the aldehyde group of 2-FPBA and the primary amine within the PU matrix to generate imine bonds (C=N, 1663 cm^−1^) [39] and (2) the borate bond in 2-FPBA with the hydroxyl group in PU to generate borate ester bonds (B-O, 1305 cm^−1^) [40] (Figure 3D). Since these two covalent bonds are highly dynamically reversible, the elastomer can proceed spontaneously at room temperature without additional stimulation.

Since both MXene and GO have excellent near-infrared (NIR) responsiveness, MG was incorporated into the PP matrix as a photothermally conductive filler. Under 0.14 W irradiation, MGPP could gradually warm and stably maintain at 40 °C within 120 s [41] (Figure 3F). When the power is increased to 0.39 W, MGPP stabilizes at 60 °C within 60 s. The thermal energy generated by NIR absorption could accelerate the healing rate resulting in rapid diffusion and re-entanglement at the fractured interface [34]. These results suggest that dynamic bond exchange and intermolecular diffusion of polymer chains at the interface give PP and MGPP excellent self-healing properties [34,35].

**Figure 4:**
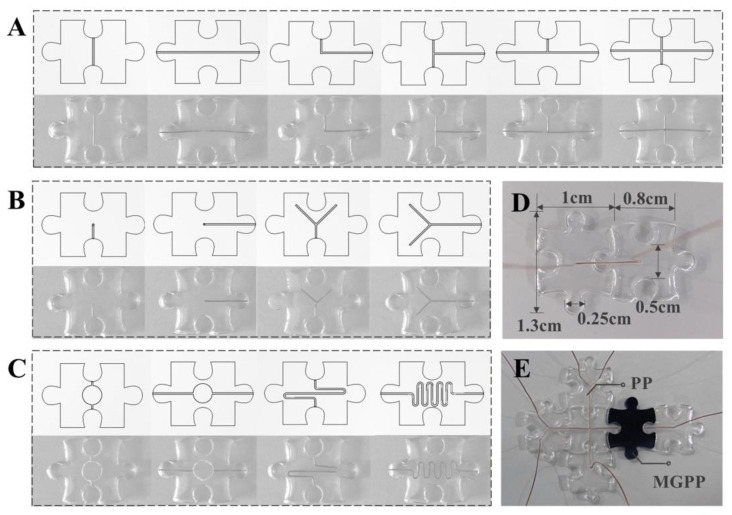
Modular microfluidic design. It includes the design of basic modules for (**A**) connecting flow channels (I/L/T/+ type flow channels) and (**B**) inlet/outlet modules (I type/Y type), and (**C**) functional modules (mixing module, reservoir/reaction chamber, electrochemical detection module). The electrochemical detection module is formed by replacing the PP that forms the reservoir/reaction chamber with MGPP. (**D**) Ink injection to check for leaks. (**E**) Microfluidic electrochemical biosensing platform.

The excellent self-healing ability of PP and MGPP makes it possible to become a modular microfluidic. Modular components can quickly realize different shapes and channels of varying precision by designing and manufacturing different molds for casting [42]. To enable fast positioning and smooth assembly between modules, we designed it as a universal puzzle shape that can be easily assembled and reconfigured intuitively [7]. Moreover, various microfluidic modules have been designed for standard application requirements. These include I/L/T/+ type flow channels (Figure 4A), I/Y type inlet/outlet modules (Figure 4B), mixing modules, reservoir/reaction chambers, and electrochemical detection modules (Figure 4C). Since self-healing may lead to collapse and blockage of the fluid channels and affect their accuracy [12], the ink is infused into their inlets to show their structure clearly. The absence of observable leakage at the interface (Figure 4D) demonstrates the self-healing strategy’s reliability and the potential of modular components for fabricating complex structured microfluidic chips [42].

Microfluidic modules are produced cost-effectively through injection molding and non-destructive demolding. Moreover, the self-healing properties allow for rapid integration and assembly in multi-planar, functionally complex networks without requiring specialized modular interfaces. The standardized modular component library developed here is, therefore, expected to enable mass production and application [7]. The modular microfluidic can, theoretically, be extended to almost infinite lengths. However, it is worth noting that this approach can reverse the connections up to 2–3 times. Since the module is disposable in the presented application, the number of possible reconnections is acceptable. In particular, the simplicity and ease of the self-healing strategy allows anyone, including non-experts, to build microfluidic platforms quickly.

Thus, we selected several modules, including an electrochemical module, to build a microfluidic electrochemical biosensing platform (Figure 4E). We investigated whether the platform could achieve stable and highly sensitive detection of colorectal cancer exosomes [43]. Specifically, we used MGPP as a self-supporting electrode. TDN containing Epcam aptamer will be immobilized on Au nanoparticles (AuNPs) via Au-S bonds [2]. Theoretically, the TDN will act as a recognition molecule to capture the exosomes. It relies on its binding to the protein overexpressed on the exosome membrane. The targeted immobilization of the aptamer can significantly improve the availability of the suspended exosomes [2]. With the presence of exosomes, quantitative differences in currents achieve the quantitative detection of exosomes [43].

**Figure 5:**
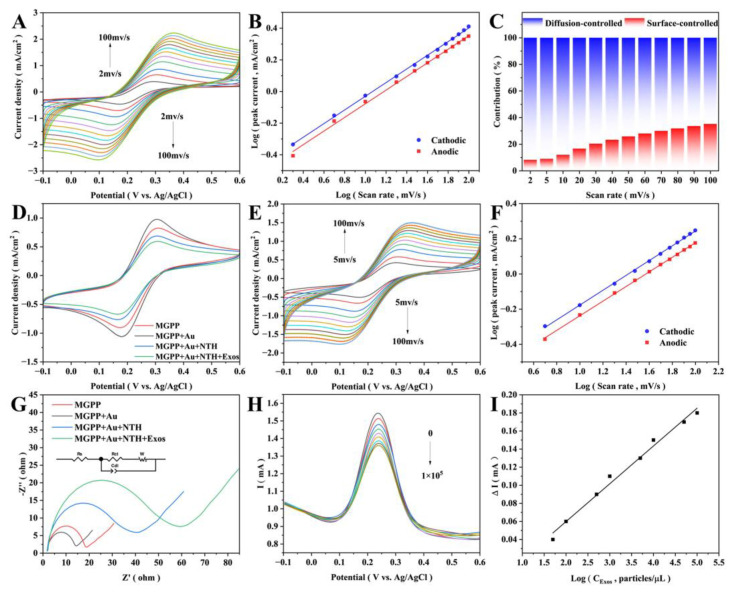
Construction of electrochemical biosensor and quantitative detection of exosomes. Self-supported electrode’s (**A**) CV curves at different sweep speeds (from top to bottom: 2, 5, 10, 20, 30, 40, 50, 60, 70, 80, 90, 100 mV/s), (**B**) relationship between the sweep speed log and peak current log, and (**C**) contribution of surface control and diffusion control to the current at different sweep speeds. (**D**) CV of the electrochemical biosensor assembly process. Assembled sensor’s (**E**) CV curves at different sweep speeds (from top to bottom: 5, 10, 20, 30, 40, 50, 60, 70, 80, 90, 100 mV/s) and (**F**) relationship between the sweep speed log and the peak current log. (**G**) EIS of the sensor assembly process, inset shows the fitted equivalent circuit diagram. (**H**) DPV of the sensor detecting different concentrations of exosomes (from top to bottom: 0, 50, 10^2^, 5 × 10^2^, 10^3^, 5 × 10^3^, 10^4^, 5 × 10^4^, 10^5^ particles/μL). (**I**) Relationship between the exosome concentration’s log and the peak currents’ difference.

We first explored the electrochemical characteristics of MGPP as a self-supporting electrode. As the sweep speed increased, the CV curve did not deform significantly (Figure 5A), indicating a low internal resistance. Moreover, the peak current was proportional to the square root of the sweep speed (Figure 5B), and the slopes of both the cathode and the anode peaks tended to 0.5, indicating that a diffusion-controlled electrochemical process occurred on the electrode surface [44]. The contribution of each component to the current was further quantified, and the surface control contribution accounted for 12% of the total current at 10 mV/s (Figure 5C). As the sweep speed increased, the surface control contribution increased correspondingly from 8% to 35% of the total current, indicating that the electron transfer process was dominated by diffusion-controlled behavior [44].

We then examined the CV (Figure 5D) and EIS behavior (Figure 5G) of the electrochemical biosensing platform during construction [45,46,47]. The change in current response is associated with the impeded electron transfer kinetics [48]. The AuNPs have a significant catalytic effect on the redox reaction of ferricyanide, which leads to an increase in CV current and a decrease in the electron transfer resistance (Ret) [49]. The phosphate backbone of TDN and the surface of the exosome are negatively charged. Therefore, it will be challenging to approach the electrode surface due to the electrostatic repulsion of ferricyanide. The decrease in CV peak current and the increase in Ret confirm the immobilization of TDN and the specific binding to exosomes [50]. Together, these indicate a successful assembly of the electrochemical biosensor. The peak current showed an excellent linear relationship with the square root of the sweep speed in the sweep speed range of 5 mV/s–100 mV/s (Figure 5E,F), indicating that the diffusion process mainly controls the electron transfer process of the assembled sensor [44].

Figure 5H demonstrates the DPV behavior of the electrochemical sensing platform at different concentrations of exosomes. The DPV current decreases with increasing exosome concentration due to TDN blocking some channels for electron/ion transport between the electrode and the electrolyte [46,49]. The peak DPV current shows an excellent linear relationship with the log value of the concentration at exosome concentrations of 50–10^5^ particles/μL [50,51] (Figure 5I). The linear regression equation was ΔI = 0.0418logC_Exos_−0.0238 (R^2^ = 0.989), and the LOD was 42 particles/μL (S/N = 3). This indicates that the sensor has a high sensitivity [52,53].

**Figure 6:**
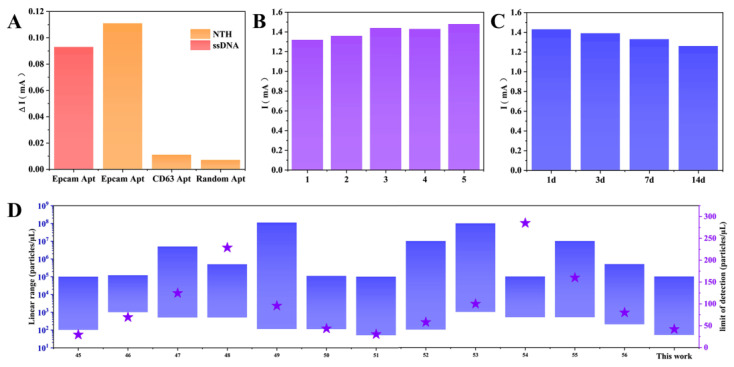
Performance and Comparison of Electrochemical Biosensors. (**A**) Selectivity, (**B**) stability, and (**C**) repeatability of the sensor. (**D**) Comparison of this work with published articles on the detection of exosomes. The blue area represents the detection range, and the purple five-pointed star represents the LOD.

Changing the single-stranded DNA (ssDNA) of the constructed NTH from Epcam aptamer to CD63 aptamer and random aptamer, a limited signal response was observed (Figure 6A) on the electrodes due to the inability of exosomes to be stably captured on the electrodes [43,54], confirming a reasonable specificity. Since ssDNA undergoes local aggregation or entanglement, the low sensitivity of single-stranded aptamers to TDN. The 4.59% relative standard deviation (RSD) obtained for the five manufactured independent sensors indicates acceptable inter-batch reproducibility [52,55] (Figure 6B). Notably, this RSD also includes the manufacturing variation of each electrode, so the obtained RSD values are outstanding. The stability of the proposed platform was periodically evaluated by recording the DPV current response. The peak DPV signal decreased slightly after the sensor was stored at 25 °C for 14 days (Figure 6C), retaining 88% of the initial peak current, indicating the high long-term stability of this sensor [56]. Figure 6D compares the performance between previous papers and present studies [45,46,47,48,49,50,51,52,53,54,55,56]. Our microfluidic electrochemical biosensing chip shows significant improvement or equivalent performance in the detection range and lower limits. In addition, our platform shows clear advantages and advances [43], such as developing a sensing platform that can be quickly assembled to obtain a microfluidic system that meets the user’s experimental needs, significantly lowering the threshold for using microfluidic technology.

## 4. Conclusions

Here, we demonstrate a conductive elastomeric MGPP with self-healing properties by combining a metal-in-situ reduced MG conductive network with a hard room-temperature self-healing elastomeric substrate PP. At the same time, we propose a strategy to use the self-healing properties for modular microfluidics, where a simple assembly method without external interfaces allows anyone to perform microfluidic engineering efficiently. The embedding of GO between MXene layers allows MG to have a larger layer spacing (1.71 nm) than MXene, effectively preventing the self-accumulation of rGO or MXene nanosheets. The stable layer spacing of MG is mainly attributed to the formation of Al-O bonds. The highly dynamic and reversible exchange of covalent bonds (imine and borate bonds) and the intermolecular diffusion of polymer chains at the interface allow PP to proceed spontaneously at room temperature without additional stimulation. Higher temperatures accelerate the healing rate of PP (12 h at room temperature to 1 h at 60 °C). We have designed several basic modules (I/Y inlet/outlet modules, I/L/T/+ flow channels) and functional modules (mixing modules, reservoir/reaction chambers, and electrochemical detection modules). The precisely positionable puzzle shape and excellent self-healing capability significantly improve the connectivity of the modules. As a proof of concept, we used DNA tetrahedra containing Epcam aptamer as recognition elements and selected suitable modular components to constitute the microfluidic electrochemical biosensor. The device has a wide detection interval (50–10^5^ particles/μL) and high sensitivity (42 particles/μL) (S/N = 3), demonstrating its potential to become a disposable and convenient integration platform.

Overall, modular microfluidics based on self-healing conductive elastomers is demonstrated here. To our knowledge, this is the first exploration of self-healing conductive elastomers for a modular microfluidic electrochemical biosensing platform. In future work, the scalable modular microfluidic devices will be further enhanced (e.g., separation modules, cell modules, flow resistance modules, droplet modules, and magnetic suction modules) in order to make the library of modular components larger and better suited to the needs of complex microfluidic system design. The proposed approach may be a versatile tool for fabricating conventional and next-generation multifunctional microfluidic structures and devices. It could provide a convenient platform for studying biomedical applications such as disease diagnosis, drug screening, cell culture, organ-on-a-chip, droplet generation, and bio-scaffolds.

## Data Availability

The data presented in this study are available on request from the corresponding author.
